# A Perilous Combination: *Streptococcus* Coinfection with Human Plague—Report of Two Cases and Review of the Literature, 1937–2022

**DOI:** 10.1089/vbz.2022.0084

**Published:** 2023-07-11

**Authors:** Brian Erly, Shannon Fleck-Derderian, Katharine M. Cooley, Kim Meyer-Lee, Jennifer House, Elizabeth VinHatton, Christina A. Nelson

**Affiliations:** ^1^ Colorado Department of Public Health and Environment, Denver, Colorado, USA.; ^2^ Division of Vector-Borne Diseases, National Center for Emerging and Zoonotic Infectious Diseases, Centers for Disease Control and Prevention, Fort Collins, Colorado, USA.; ^3^ Larimer County Department of Health and Environment, Fort Collins, Colorado, USA.; ^4^ New Mexico Department of Health, Santa Fe, New Mexico, USA.

**Keywords:** *Yersinia pestis*, Streptococcus, coinfection, plague

## Abstract

**Background:**

Plague in humans and animals is caused by *Yersinia pestis*, a zoonotic gram-negative bacterium endemic in certain regions of Asia, Africa, and the United States. Coinfection with both *Y. pestis* and Streptococci species has been anecdotally reported in humans and associated with severe and rapidly fatal disease.

**Methods:**

This report presents two cases of patients who died following *Y. pestis* and *Streptococcus* coinfection. Additional cases of previously published *Y. pestis–Streptococcus* coinfection were identified and reviewed using a search of electronic databases.

**Results:**

The first case patient developed cough and dyspnea following 4 days of fever, malaise, and back pain and died before receiving medical care. Postmortem blood cultures were positive for *Y. pestis*, *Streptococcus pyogenes*, and *Streptococcus dysgalactiae*. The second case patient was hospitalized with fever, vomiting, diarrhea, and dyspnea and died of sepsis and respiratory failure on the day of admission. *Y. pestis* and *Streptococcus pneumoniae* were isolated from blood cultures drawn on admission. Seven additional cases of *Y. pestis* and *Streptococcus* coinfection were identified, dating between 1948 and 2009. These patients were healthy overall before their illness, with ages ranging from 9 to 60 years. The majority of patients had primary bubonic plague with associated pneumonia or septicemia. None of the patients who died received timely antimicrobial therapy directed against gram-negative pathogens. In every case but one, an occupational or environmental risk factor for plague was later identified.

**Conclusion:**

*Y. pestis* infection begins with a pre-inflammatory phase, during which *Y. pestis* and other pathogens can rapidly proliferate. Streptococci, which are frequently asymptomatic colonizers, may become invasive in this environment, leading to coinfection. The challenges of diagnosing *Y. pestis* in the context of coinfection may delay effective treatment. This case series and literature review illustrate the importance of clinicians remaining alert to environmental and occupational exposures in patients presenting with an infectious syndrome, especially in those who have an unexpectedly severe clinical presentation.

## Introduction

*Y**ersinia pestis* is a gram-negative bacterium that is the causative agent of plague, a zoonotic infection transmitted primarily by flea bites. Reservoirs of *Y. pestis* are found on all continents except Australia and Antarctica (Mead, 2015). In the western United States, *Y. pestis* is maintained in animal reservoirs and can occasionally cause outbreaks among small mammals, including prairie dogs, squirrels, and other ground-dwelling rodents (Barbieri et al., 2020). Plague is rare in the United States, with only 468 human cases reported from 1965 to 2012, the majority of which occurred in the Southwest. The median age of afflicted patients is 28 years, and there is a slight male predominance (57%) (CDC, 2021; Kugeler et al., 2015).

An important clinical feature of plague is the rapid transition from a “pre-inflammatory phase,” during which the host may have minimal or nonspecific symptoms, to an overt septic phase. This rapid deterioration makes early attention to risk factors and clinical signs important, as prompt identification of the causal agent and initiation of appropriate antimicrobial therapy is critical to reduce morbidity and prevent mortality (Demeure et al., 2019; Nelson et al., 2021).

*Streptococcus* is a genus of more than 100 species of gram-positive bacteria commonly responsible for disease in both humans and animals (Nguyen et al., 2015). Group A Streptococci have surface proteins, which inhibit phagocytosis and secrete pyrogenic toxins, resulting in a massive nonspecific inflammatory response. In humans, group A *Streptococcus* can cause a range of serious invasive infections, which include pneumonia, septic arthritis, necrotizing fasciitis, and sepsis. Invasive group A *Streptococcus* infection is relatively common in the United States compared with plague, with an estimated rate of 7.63 cases per 100,000 persons in 2019. Peak incidence occurs later in life, with the highest rate among individuals older than 85 years (27.8 cases per 100,000 persons) (CDC, 2019).

*Streptococcus pneumoniae* most frequently causes respiratory infection in the context of disrupted epithelial barrier (whether due to preceding illness or other host factors), but the bacteria can also cause systemic illness, which is facilitated by a polysaccharide capsule (Wright and Myers, 2019). Nutritionally variant streptococci are normal components of human oropharyngeal, gastrointestinal, and urogenital flora but may cause endocarditis and other invasive infections in older patients and those with indwelling devices (Alberti et al., 2016).

In general, the presence of bacterial coinfections among patients may delay or complicate appropriate diagnosis and treatment and worsen patient outcomes. Moreover, anecdotal reports suggest that coinfection specifically with *Y. pestis* and *Streptococcus* spp. can increase severity and lead to rapidly progressive disease; however, to the best of our knowledge, this phenomenon has not been systematically evaluated. In this report, we present two patients with *Y. pestis* and *Streptococcus* coinfection who experienced rapid deterioration and death. We also review available published case reports of *Y. pestis–Streptococcus* coinfection.

## Methods

Investigation of the cases described in this report was completed jointly by the Centers for Disease Control and Prevention (CDC), Colorado Department of Public Health and Environment, Larimer County Department of Health and Environment, and the New Mexico Department of Health. Local, state, and federal public health agencies worked to review medical records, interview family members, and conduct an environmental investigation.

Other reports of infection with *Y. pestis* and *Streptococcus* were identified through a systematic literature review on antimicrobial treatment of plague (methods detailed elsewhere) (Nelson et al., 2020). Briefly, articles were identified through database keyword search of Cochrane Library, ClinicalTrials.gov, the Cumulative Index to Nursing and Allied Health Literature, the Defense Technical Information Center, EBSCO Global Health, Embase, Medline, PubMed Central, and Scopus from database inception to July 2022 to identify indexed publications containing search terms related to plague and antimicrobial treatment. Published articles describing patients with documented *Y. pestis* and *Streptococcus* coinfection were flagged for further review. Additional case reports were identified via reference review in articles describing plague treatment.

### Study selection

We included any publication that contained case-level information on a patient diagnosed with plague and a *Streptococcus* coinfection. Non-English articles that potentially included case-level data were professionally translated and included if they met the inclusion criteria.

### Data abstraction and analysis

Information from each included article was manually abstracted and summarized. Data were collected on patient demographics, exposure history, incubation period, time from symptom onset to medical evaluation, signs and symptoms, laboratory testing, treatment, complications, and outcome.

For this review, the primary clinical form of plague for each patient was defined as the initial presenting manifestation (bubonic, pneumonic, etc.). Secondary clinical forms of plague were defined as any subsequent manifestation(s).

## Results

### Case report: Colorado—2015

A previously healthy 16-year-old male from rural Colorado experienced fever, malaise, and lower back pain. On the fourth day of illness, he developed a cough and difficulty breathing. In the early morning hours of the following day, after an episode of hemoptysis, he was transported to the hospital but died of cardiac arrest *en route*.

PCR testing of bloody sputum collected before the patient's death was positive for *Y. pestis*. Blood and lung cultures were positive for group A *Streptococcus* (*Streptococcus pyogenes*) and *Y. pestis*. 16S sequencing of Streptococci isolated from blood identified two different species, *S. pyogenes* and *S. dysgalactiae* subsp. *equisimilis*.

Postmortem examination revealed no apparent arthropod bites or other skin lesions. There was no evidence of prior illness or injury and no visible bubo. The pulmonary parenchyma had areas of edema, exuding large amounts of frothy fluid, without focal lesions or obvious consolidation. Green mucoid material was present on pleural surfaces and extended throughout the fissures of the lungs. Pleural and pericardial serous effusions were present, along with cardiomegaly and splenomegaly.

Microscopic examination of the lung tissue revealed a mixed inflammatory infiltrate with mononuclear predominance. This was also evident on the pleural surface, which appeared thickened. Areas of pulmonary edema were also present. Dark rod-like bacteria were seen in vascular structures associated with some expanded fissures. No well-developed bronchopneumonia was present.

The myocardium had one focus of wavy fiber change, immature fibrosis, and mononuclear infiltrates associated with cocci-shaped bacteria. A possible focus of myocyte necrosis was also seen in this area. The spleen was congested, with reactive mononuclear cells in the sinuses.

Environmental investigation revealed that two of the patient's four dogs were seropositive for *Y. pestis*, indicating that exposure likely occurred at or near the patient's residence. The patient was an avid hunter and had direct contact with prairie dogs and other wild animals before his illness.

### Case report: New Mexico—2015

A 52-year-old woman from Santa Fe County, New Mexico, developed chills, sweats, vomiting, and diarrhea and received medical care 2 days later for presumed gastroenteritis. Two days after this, she was hospitalized with fever, chills, anorexia, abdominal pain, vomiting, diarrhea, dyspnea, and tachycardia. White blood cell count was 56.5 × 10^3^/μL; laboratory test results also indicated signs of acute kidney injury. No buboes, insect bites, or skin lesions were noted. Chest X-ray revealed extensive ill-defined opacities bilaterally, consistent with pneumonia. The patient was given one dose of broad-spectrum antibiotics for presumed sepsis; however, she did not receive an antibiotic considered highly effective for plague treatment (aminoglycoside, fluoroquinolone, or tetracycline class antibiotic).

Six hours after admission, the patient developed worsening sepsis and acute respiratory failure and died. Primary septicemic plague with secondary pneumonic plague was considered the cause of death. *Y. pestis* and *S. pneumoniae* were ultimately isolated from blood cultures drawn on admission.

The patient had no recent history of travel but had been camping at Fort Sumner, New Mexico, on the day of illness onset. She lived in a mobile home in an area where numerous rodents were also noted to reside. A dead rabbit was found in the patient's yard ∼1 week before her illness onset. Approximately 1–2 weeks before the patient's illness, her cat was reported to be ill and lethargic for several days. Subsequent investigation revealed that the cat had had confirmed plague, with a ≥4-fold change in serological titer for *Y. pestis*.

### Literature review

Seven additional cases of *Y. pestis–Streptococcus* coinfection were identified during the literature search. These included one case of primary pneumonic plague, two of septicemic plague, and four of bubonic plague with concomitant *Streptococcus* infection. One patient with bubonic plague developed secondary pneumonic plague. Four patients had coinfection with *S. pneumoniae*, two with group A *Streptococcus*, one with β-hemolytic *Streptococcus* (not further specified), and one with nutritionally variant *Streptococcus*. The median age of patients was 31 years (range 9–60). One patient recovered (14%), whereas all other patients died. Cases are summarized in Table 1.

**Table 1.** Summary of Cases of *Yersinia pestis*–*Streptococcus* Coinfection

Source	Coinfection	Year	Patient age; known comorbid conditions	Plague exposure	Clinical manifestation	Antimicrobial therapy	Outcome
Feng (1949)	*Streptococcus pneumoniae*	1948	31	Laboratory	Primary pneumonic. Note: patient had previously received a plague vaccine	Sulfadiazine; Penicillin; Streptomycin; Anti-plague serum	Recovered
Mengis (1962)	Group A β-hemolytic *Streptococcus*	1961	38	Outdoor activities	Primary bubonic; secondary pneumonic and septicemic	None	Died
CDC (1964)	*S. pneumoniae*	1963	28	Rabbits	Primary bubonic; secondary pneumonic and septicemic	None	Died
Jones et al. (1979)	β-hemolytic *Streptococcus* (not further identified)	1976	15	Chipmunk	Primary bubonic; secondary pneumonic and septicemic	Penicillin	Died
Maehara et al. (1983)	Group A β-hemolytic *Streptococcus*	1983	9	Household pet	Primary bubonic; secondary septicemic	Penicillin	Died
CDC (1997), Levy and Gage (1999)	*S. pneumoniae*	1996	16	Unknown	Primary bubonic; secondary septicemic and meningitic	Ceftriaxone	Died
CDC (2011)	Nutritionally variant *Streptococcus*	2009	60; Insulin-dependent diabetes mellitus	Laboratory	Primary pneumonic; secondary septicemic	Vancomycin, piperacillin/tazobactam	Died
Present report	*Streptococcus pyogenes* and *Streptococcus dysgalactiae* subsp. *equisimilis*	2015	16	Outdoor activities near residence	Primary septicemic; secondary pneumonic	None	Died
Present report	*S. pneumoniae*	2015	52	Pet cat	Primary septicemic; secondary pneumonic	One dose of broad-spectrum antibiotics; did not receive antibiotics considered effective for plague	Died

#### Case 1: Fuzhou, China—1948

A 31-year-old man who worked at the Fukien Provincial Health Laboratory developed malaise and fatigue, followed a day later by hemoptysis. He had received two doses of plague vaccine the prior year. The patient was hospitalized the following day and noted to be febrile, tachypneic, and tachycardic. He was treated with sulfadiazine based on suspicion of plague, which was continued when the diagnosis was confirmed with sputum culture collected the same day. Penicillin was also started as prophylaxis against gram-positive infections. The patient's clinical condition improved on the fifth day of hospitalization. Serial sputum culture revealed *S. pneumoniae* on the 10th day after infection. On the 15th day after symptom onset, the patient experienced a return of fever, which resolved the following day; he was discharged 16 days after onset (Feng, 1949).

#### Case 2: Santa Fe, New Mexico—1961

A male geologist, aged 38 years, investigated ground movement in arroyos (drainage ditches) around Santa Fe 1 day before developing a small sore on the back of his thumb. Four days later, he developed fatigue, fever, and axillary tenderness and lymphadenopathy. He experienced a period of improved symptoms before developing fever and nausea on the sixth day after symptom onset. On laboratory analysis, his white blood cell count was 25,000, with 88% neutrophils; due to his reassuring examination, plans were made to defer admission until the next morning. However, on the evening of the sixth day after symptom onset, he was brought to the hospital by ambulance due to hemoptysis and shortness of breath. By that time, he had developed a generalized petechial rash and the sore on his hand had developed a black eschar at the base. The patient died in the hospital before antimicrobial therapy could be initiated. Blood cultures drawn shortly before death revealed *Y. pestis* and group A *Streptococcus* (Mengis, 1962).

#### Case 3: Houck, Arizona—1964

A 28-year-old male shepherd developed sudden onset of chills, headache, abdominal pain, diarrhea, and bloody vomit, along with axillary pain. The patient habitually prepared and consumed rabbits for food, and had been grazing his sheep for 5 days before the onset of symptoms. The patient was driven to the hospital 2 days after symptom onset. His initial examination was remarkable only for tachycardia and abdominal tenderness with guarding but no rebound. He was admitted to the hospital with suspicion of intra-abdominal pathology requiring surgery. However, later that day, he developed hypotension and tachypnea, and axillary adenopathy was noted. Leukocytosis and gram-negative rods were noted on blood smear. He was then started on streptomycin and chloromycetin but died that same day, 2 days after symptom onset. Premortem blood cultures revealed *Y. pestis* and *S. pneumoniae* (CDC, 1964).

#### Case 4: Albuquerque, New Mexico—1976

A 15-year-old boy went on a picnic in the mountains near Albuquerque, New Mexico, where he had contact with a chipmunk. The following day he presented to the local emergency room with fever, headache, and malaise, where a throat culture was collected. He received unspecified treatment and was released. The next day he went to the hospital with worsening fever, headache, and malaise and was again sent home. A tender inguinal mass appeared the following day (after symptom onset day 2); he returned to the hospital and was admitted on post-onset day 3.

In the hospital, enlarged inguinal lymph nodes were noted. As the previously collected throat culture was positive for β-hemolytic streptococci, the patient was given oral penicillin, and a blood culture was taken. The patient continued to experience fever and malaise, and a biopsy of the enlarged nodes revealed gram-negative bacilli. He was then treated with streptomycin on suspicion of plague. Later that day, on post-onset day 4, the patient developed signs consistent with diffuse intravascular coagulation and gastrointestinal hemorrhage, and he died (Jones et al., 1979).

#### Case 5: Nevada—1983

A 9-year-old boy from northern Nevada was admitted to the hospital following several days of vomiting and abdominal pain. On admission, physical examination was notable for fever, tachycardia, and supraclavicular pain. Laboratory tests were remarkable for leukocytosis, and throat culture revealed group A *Streptococcus*. The patient was started on penicillin. The following day, he developed shock and died despite resuscitative efforts. Cerebrospinal fluid culture grew group A *Streptococcus*, and blood cultures revealed both group A *Streptococcus* and *Y. pestis* (Maehara et al., 1983).

#### Case 6: Colorado—1996

A 16-year-old girl from Colorado developed sudden left arm numbness and left axillary pain. The following day, she developed fever, chills, and vomiting. On post-symptom onset day 2, she presented to the emergency department where she was tachycardic, but other vital signs were within normal limits and chest X-ray was normal. Her symptoms were attributed to a mechanical injury several days prior causing brachial plexus injury, and she was prescribed analgesics and discharged for outpatient follow-up.

On the fourth day after symptom onset, she was found semiconscious at home and brought to the hospital. On admission, she was confused and complained of neck pain and generalized soreness. She was febrile, tachypneic, and tachycardic. She developed respiratory arrest and was intubated, and gram-positive diplococci were noted on blood smear. She was given ceftriaxone and transferred to a referral hospital for further treatment. However, she died later that day. Blood cultures later revealed *S. pneumoniae* and *Y. pestis*. Later investigation revealed a prairie dog die-off near the patient's home and high antibody titers to *Y. pestis* in 4/5 family dogs and 1/3 family cats (CDC, 1997; Levy and Gage, 1999).

#### Case 7: Chicago, Illinois—2009

A 60-year-old laboratory researcher with type 1 diabetes presented to an outpatient provider following 3 days of fever, body aches, and cough. The patient worked with an attenuated *Y. pestis* strain. He was referred to the emergency department but did not seek further care until 3 days later (post-symptom onset day 6) when he was brought to the hospital by Emergency Medical Services due to fever, cough, and shortness of breath. He was febrile, tachypneic, and tachycardic on arrival. No lymphadenopathy was noted on admission, although blood work showed leukocytosis. The patient was treated with intravenous vancomycin and piperacillin/tazobactam, as well as diuretics for suspected congestive heart failure. He developed respiratory failure and was intubated, developed cardiac arrest 1 h later, and was not able to be resuscitated. Blood cultures drawn on admission revealed both nutritionally variant *Streptococcus* and a pigmentation-negative (pgm−) attenuated strain of *Y. pestis*. Postmortem examination revealed previously undiagnosed hemochromatosis.

Before his illness, the patient worked in a university laboratory with a pgm− *Y. pestis* strain (KIM D27). pgm− *Y. pestis* lacks 11 genes involved in iron uptake during mammalian infection and is excluded from the National Select Agent Registry (Federal Select Agent Program, 2020). However, in murine models, pathogenicity is restored following infusion of inorganic iron (Lee-Lewis and Anderson, 2010). Iron overload due to hemochromatosis may have rendered the patient susceptible to this normally attenuated strain (CDC, 2011).

## Discussion

In this review, we describe nine cases of plague complicated by coinfection with *Streptococcus* spp. In all but one of these cases, infection with both *Y. pestis* and the second pathogen appeared to occur simultaneously. In these cases, the patients did not receive timely and effective antimicrobial treatment and died from rapidly progressive infection. These findings illustrate the importance of early identification of clinical syndromes consistent with plague and prompt initiation of appropriate antibiotics.

The single case from Fuzhou, China, in which the patient survived, is distinct from the others in at least four important ways—the patient had been vaccinated against *Y. pestis*, he received effective antimicrobial treatment against *Y. pestis* shortly after symptom onset, additional therapy was given to guard against bacterial coinfection, and the apparent onset of the second bacterial infection was delayed relative to the initial presentation. Timely effective antimicrobial therapy (rather than ineffective or no treatment) along with a superinfection pattern (rather than coinfection) likely played a role in the recovery of the patient (Huang and Huang, 1948). All other reported cases resulted in death, despite the patients being largely without significant medical comorbidities apart from a single case with type 1 diabetes.

Simultaneous infection with multiple pathogens can result in a more severe disease process, in addition to obfuscating the overall clinical picture (McArdle et al., 2018). *Y. pestis* has several unique features, which may make coinfection more common; in pneumonic plague, the pre-inflammatory stage may allow for proliferation of both *Y. pestis* and other pathogenic organisms in the lungs (Price et al., 2012). This pre-inflammatory phase involves activation of the proinflammatory cytokines IL-1β and IL-18, which is balanced early in the disease course by induction of anti-inflammatory protein IL-1 receptor antagonist (Sivaraman et al., 2015). IL-1β signaling is involved in the immune response to multiple species of streptococci, and IL-1β-deficient mice are more susceptible to streptococcal infections (LaRock and Nizet, 2015). Simpson's (1905) “Treatise on Plague” stated, “It is not infrequent to meet with a mixed infection in plague, and in these cases the pneumococcus may be found with the plague bacillus …”

There are also additional descriptions of *Y. pestis* coinfection with nonstreptococcal pathogens. Case reports of multidrug-resistant *Stenotrophomonas maltophilia* and leptospirosis coinfection with plague have been previously published, and a genomic study of a sixth-century plague victim showed evidence of invasive *Haemophilus influenzae* (serotype b) (Andrianaivoarimanana et al., 2018; Bertherat et al., 2014; Guellil et al., 2022). However, based on the available case data, *Y. pestis–Streptococcus* coinfection appears to carry a greater risk of mortality than other pathogens in combination with *Y. pestis*.

The poor outcomes associated with *Y. pestis* and *Streptococcus* coinfection are likely due to at least one of two potential mechanisms. First, in many of the reviewed cases, the *Streptococcus* infection was more readily detected than the *Y. pestis* coinfection, perhaps resulting in premature diagnostic closure and ineffective antibiotic treatment (Graber et al., 2005). Untreated bubonic plague has a mortality rate of 50–90%; pneumonic, meningitic, and septicemic plague are fatal in most cases if untreated (Prentice and Rahalison, 2007). Second, mortality may be increased by the diverse immunomodulatory and immune escape strategies employed by two distinct causal organisms, resulting in synergistic immune dysregulation and a more rapidly progressive infectious syndrome (Nguyen et al., 2015). Additional experimental studies to elucidate this relationship would be helpful.

This case series has some notable limitations. The series is limited to cases in the available literature, along with those reported to CDC. While every effort was made to identify all cases in the extant literature, unpublished cases and cases in which the coinfection was not detected are excluded. As a result, this report might be biased toward more severe cases. Similarly, cases where initial empiric antibiotic therapy successfully treated one or both pathogens may have resulted in an unrecognized coinfection. Conversely, it was not possible to rule out specimen contamination or colonization with *Streptococcus* in the context of an underlying *Y. pestis* infection, rather than true coinfection. Finally, information on published cases was limited, and direct review of medical records was not possible.

To prevent avoidable morbidity and mortality associated with *Y. pestis*–*Streptococcus* coinfection, clinicians should remain alert to environmental and occupational exposures in all patients presenting with an infectious syndrome, especially in those who have an unexpectedly severe clinical presentation or rapid deterioration. In many of the cases described here, tender swollen lymph nodes with a compatible environmental exposure could have suggested bubonic plague.

Patients with *Y. pestis* and *Streptococcus* coinfection present a clinical challenge given the severity of illness, relative rarity of plague, and presence of a second pathogen with many overlapping clinical features. Prompt initiation of empiric antimicrobial therapy directed against the spectrum of likely pathogens, including zoonotic organisms, may help improve outcomes in patients affected by this severe coinfection. When plague is suspected, providers should observe treatment guidelines, including initiating therapy with two classes of antimicrobials for patients with severe pneumonic or septicemic plague (Nelson et al., 2021).
